# Are Thai MSM Willing to Take PrEP for HIV Prevention? An Analysis of Attitudes, Preferences and Acceptance

**DOI:** 10.1371/journal.pone.0054288

**Published:** 2013-01-14

**Authors:** Ana Wheelock, Andreas B. Eisingerich, Jintanat Ananworanich, Gabriela B. Gomez, Timothy B. Hallett, Mark R. Dybul, Peter Piot

**Affiliations:** 1 Centre for Patient Safety and Service Quality, Imperial College London, London, United Kingdom; 2 Imperial College Business School, Imperial College London, London, United Kingdom; 3 The Thai Red Cross AIDS Research Centre and Faculty of Medicine, Chulalongkorn University, Bangkok, Thailand; 4 Department of Global Health, Academic Medical Centre, University of Amsterdam and Amsterdam Institute for Global Health and Development, Amsterdam, The Netherlands; 5 Department of Infectious Disease Epidemiology, Imperial College London, London, United Kingdom; 6 Georgetown O’Neill Institute for National and Global Health Law and the George W. Bush Institute, Washington, D.C., United States of America; 7 London School of Hygiene and Tropical Medicine, London, United Kingdom; Vanderbilt University, United States of America

## Abstract

**Objective:**

We aimed to understand the attitudes, preferences and acceptance of oral and parenteral PrEP among men who have sex with men (MSM) in Thailand.

**Background:**

Pre-exposure prophylaxis (PrEP), the use of antiretrovirals to prevent HIV acquisition, has shown promising results in recent trials. To assess the potential impact of this new HIV prevention method, in addition to efficacy data, we need to understand which psychosocial factors are likely to determine its uptake among members of potential user groups.

**Methods and Findings:**

Surveys of willingness to use PrEP products were administered to MSM. Spearman’s rank tests were used to uncover associations between questionnaire items. Mann-Whitney tests were performed to ascertain differences between groups. Conjoint analysis was used to examine the attitudes and preferences of MSM towards PrEP attributes. Most participants were willing to consider taking PrEP (39.2% “yes, definitely” and 49.2% “yes, probably”) and perceived PrEP as giving them new possibilities in their lives (38.5% “a lot of hope” and 55.8% “some hope”), even after being instructed of potential side effects and costs. HIV testing was considered the most important attribute and a daily pill and longer lasting injection in the arm were the preferred routes of administration.

**Conclusions:**

Despite its multiple challenges, MSM in Thailand would be willing to take PrEP, even if they had to experience inconvenience and expense. If PrEP were to be implemented in Thailand, our findings show that its uptake could be considerable.

## Introduction

In the 1990s, Thailand achieved a significant reduction in overall HIV incidence by enforcing condom use in brothels and ensuring wide access to HIV prevention information through the mass media, schools and the workplace [1], [2], [3], [4]. Despite these efforts, certain populations remain disproportionally affected by the epidemic, notably men who have sex with men (MSM). Recent surveys show that one in four MSM is infected with HIV in Bangkok [5], [6], and one in eight in Chiang Mai [7]. Improving and expanding existent HIV prevention programs, and introducing new initiatives that take into consideration the needs of this population, are urgently needed to address this health inequality.

Antiretroviral-based prevention, a strategy to prevent HIV transmission (treatment as prevention or TasP) or acquisition (preexposure prophylaxis or PrEP), could be part of the solution. Early treatment of those infected with HIV reduced the risk of transmission to uninfected partners of heterosexual couples by 96% in the HPTN-052 trial [8]. TasP, however, has yet to be tested among MSM. PrEP, on the other hand, reduced the risk of HIV infection by an average of 44% (73% among high adherers) in HIV negative MSM and transgender women who participated in iPrEx, a clinical trial testing a daily oral dose of the antiretroviral drug Truvada© [9].

Following the iPrEx results, the U.S. Food and Drug Administration recently approved the use of Truvada® for prevention purposes [10]. Clinical guidance for its use among MSM has been released in the U.S. by the Centers for Disease Control and Prevention [11] _ENREF_16and South Africa [12], and the World Health Organization has issued guidance on the use of PrEP for MSM in the context of demonstration projects [13].

As demonstration projects are beginning to take place in preparation for an eventual PrEP rollout [14], [15], multi-disciplinary research aimed at identifying where existing and new HIV prevention methods may fit best within a combination prevention package is of critical importance. An aspect which deserves consideration is users’ acceptance of the prevention methods on offer [16], [17], [18].

In the case of PrEP, emerging evidence suggests good overall acceptability among MSM, albeit with significant variance across countries. A multinational study on oral and parenteral PrEP found that 92% of MSM in India and 70% in South Africa would definitely be willing to take PrEP, whilst only 45% of MSM in Peru would definitely consider taking it [19]. Similarly, 78,5%, 74% and 44% of MSM expressed intentions to take PrEP in three different studies conducted in the U.S. [20], [21], [22], and 63% reported high willingness to use PrEP in a study conducted in China [23]. Australian MSM have thus far been the most cautious, with only 28% reporting willingness to use PrEP [24]. Importantly, these studies have shed light on country-specific factors likely to affect PrEP uptake and adherence.

This study examined the attitudes, preferences and future acceptability of real and hypothetical attributes of PrEP products among MSM in Thailand. We aimed to inform priority setting, program design, and product development, should PrEP prove cost-effective in this context.

## Methods

### Data Collection

This research was conducted between July 2011 and September 2011 in Bangkok and Chiang Mai. Our study’s methods are reported in detail elsewhere [19]. In brief, we administered a questionnaire to MSM to assess their likelihood of adopting PrEP. Ipsos MORI, a global social research company, coordinated the data collection and Ipsos Thailand carried out the fieldwork in consultation with a local NGO working with MSM and transsexuals. Questionnaire items were discussed with experienced local researchers and fieldworkers in a focus group setting to check pertinence and clarity of wording. The questionnaire was translated in Thai by the local market research team and back-translated by professional translators in London for content consistency. The final translation was agreed by consensus.

The protocol of this study was approved by the ethical committees of Imperial College London and the Institute for the Development of Human Research Protections, Ministry of Public Health in Thailand. We obtained informed written consent from all participants.

### Sample

We used targeted sampling [25] _ENREF_9to recruit participants and selected different locations to ensure a diverse sample. These included hairdressing salons, healthcare centers, hotels, non-governmental organizations (NGOs), nightclubs, red-light districts, saunas, streets, and universities. HIV prevalence is higher among MSM and male sex workers than transsexuals [26], thus MSM were prioritized.

Eligibility was determined using a brief screening interview. Inclusion criteria were self-identifying as a man who has sex with men, being 18 years old or older, self-reporting a negative or unknown HIV serostatus, being sexually active, and not having taken part in a market research study in the past 12 months. Participants received an incentive of 500 Baht (US$15) for their participation.

### Measurement

We used a combination of quantitative measures (sections 1–3 and section 5) and conjoint analysis (section 4). The questionnaire had a total of 57 items in five sections. The first four sections were interviewer-administered. Section five was self-administered due to the sensitive nature of the topics [27]. We employed verbal labels to improve data quality [28]. Unless specified here, we used four-point Likert scale items (1 = “yes, definitely”, 2 = “yes, probably”, 3 = “no, probably not”, and 4 = “no, definitely not”) to avoid midpoints, which can discourage respondents from taking a stand [29]. However, interviewers were allowed to record spontaneous “I do not know” responses.

Section 1 introduced PrEP as a medication which would reduce the risk of HIV infection in HIV negative people. A description of hypothetical and known PrEP attributes, based on the iPrEx results, expert consultations and a literature review [9], [30], [31], was provided. Participants were told that PrEP was: for people who did not have HIV; that it was ineffective against other sexually transmitted diseases; that it was being tested as a pill and eventually as an injection [31]; that clinical tests had shown that there were 73% fewer HIV infections among MSM who took daily oral PrEP most of the time – that is to say, at least nine times out of every ten – [9], that it could cause mild temporary side effects such as nausea, weight loss and headaches; and that could be partially protective against HIV, especially if not taken as directed, therefore frequent HIV tests would be needed. Participants were encouraged to ask the interviewer to repeat the description if any part was unclear. Questions about adherence to previous regular medication regimens were asked towards the beginning of this section, as a proxy measure for future adherence [32].

Section 2 explored the future acceptability and potential use of PrEP. We examined participants’ willingness to take PrEP, likelihood of early adoption, and key attitudes associated with taking PrEP: embarrassment, anxiety, hope, and fear of contracting HIV.

In section 3, we assessed potential barriers to PrEP use: side effects, cost (an affordable monthly amount equivalent to two boxes of headache tablets, as condoms are often free of charge), willingness to share and sell PrEP if given for free (a limited amount for personal use), condom use, and HIV testing.

In section 4, we elicited data for conjoint analysis, a statistical technique frequently used to determine the value people assign to different features or attributes of products or services [33], [34], to assess the relative importance of key hypothetical and known attributes of PrEP. By confronting participants with realistic tradeoff situations, conjoint analysis thus enabled us to test what combination of PrEP attributes is most critical in participants’ decision making and which attributes are most preferred. Conjoint analysis was deemed an appropriate and effective means of exploring participants’ preferences, since PrEP is a new offering and past observations (data, experience, etc.) are likely to be of limited help. More specifically, we chose attributes that represented relevant stages of an implementation program, based on discussions with academic, policy, and industry experts. Conjoint analysis was conducted as follows. First, participants were shown a card with three different PrEP scenarios depicted on it, using both graphics and text to reduce cognitive effort. Each scenario had a different combination of five attributes (and corresponding levels): (1) route of administration (a pill once a day, a pill before and after having sex, an injection in the arm once a month, or an injection in the buttocks every two months); (2) dispensing site (pharmacy, family planning clinic, health clinic, or antiretroviral treatment clinic); (3) time spent obtaining PrEP (two hours and four hours); (4) frequency of pick up (every month and every two months); and (5) frequency of HIV testing associated with PrEP (monthly or every six months). Participants then indicated their preferred choice among the three different PrEP scenarios depicted on each card, with the option to state that none of the scenarios was preferable. Each participant responded to ten different cards.

Section 5 collected demographic data, including gender, place of residence, age, and education, which we used as proxy measure for socioeconomic status [35]. Participants were then asked to disclose sensitive information to assess risk behaviors, including number of sexual partners, type of sex practiced (vaginal and anal), HIV status, condom, and drug use. Before commencing this section, participants were reminded about the strict confidentiality of their responses. Subsequently, they were given a booklet with pictorial representations of the answers to facilitate comprehension [36]. We adapted a voting box approach to reduce social desirability bias [37]_ENREF_32_ENREF_16 and asked participants to introduce the filled-out booklet in a blank envelop, seal it, and place it into a larger envelope containing other sealed booklets. Booklets had a unique code to link them back to the interviewer-administered part of the questionnaire.

### Statistical Analysis

Conventional descriptive statistics were performed to assess the characteristics of the study’s participants and their views on PrEP. Spearman’s rank tests were used to determine correlations between questionnaire items. Mann-Whitney tests were conducted to ascertain differences between groups. A *p*-value less than 0.05 was considered statistically significant. Conjoint analysis was used to examine the relative importance of key attributes of PrEP. Five attributes were used to represent PrEP scenarios. To reduce cognitive effort we combined “time spent obtaining PrEP” and “frequency of pick up”, yielding 128 possible scenarios. An efficient design of 32 scenarios was found and 120 choice tasks were generated from these 32 scenarios (by combining scenarios together into sets of three) using SAS 9.3 software. Finally, the 120 choice tasks were split into twelve blocks of ten choice tasks. Sawtooth CBC/HB Version 50.2.8 software was used to decant respondents’ choices into respondent-level utilities, using hierarchical Bayes estimation, which allowed us to determine the directionality (positive versus negative) and relative importance of each level.

## Results

### Participant Characteristics

We interviewed 260 participants; 130 in Bangkok and 130 in Chiang Mai. As reported in Table 1, the average respondent was 19–24 years old (54%), reported not having taken regular medication in the past (96%), was afraid of contracting HIV (85%), had one sexual partner in the last month (42%), had anal sex once a week in the past year (34%), and used condoms every time they had sex in the last year(48%). Most respondents reported not currently receiving gifts or money for sex (88%) and having been tested for HIV before (59%). All reported to be HIV negative.

**Table 1:** Participants’ characteristics.

CHARACTERISTIC	TOTAL
**Gender – n (%)**	
Male	259 (99)
Transgender	1 (0)
**Age group – n (%)**	
16–18 yr	11 (4)
19–24 yr	139 (54)
25–30 yr	71 (27)
31–35 yr	28 (11)
≥36 yr	11 (4)
**Education level – n (%)**	
Less than secondary	2 (1)
Completed secondary	12 (5)
Postsecondary	245 (94)
Rather not say	1 (0)
**Experience taking regular medication – n (%)**	
Having taken regular medication in the past	250 (96)
Not having taken regular medication in the past	9 (4)
Do not know/Cannot remember	1 (0)
**HIV testing – n (%)**	
Having been tested	153 (59)
Not having been tested	107 (41)
**Fear of contracting HIV**	
Very afraid	161 (62)
Fairly afraid	59 (23)
Not very afraid	22 (9)
Not at all afraid	18 (7)
**Sexual risk factors – n (%)**	
**Number of partners in the last month**	
1 partner	108 (42)
2 partners	62 (24)
3–5 partners	37 (14)
≥6 partners	13 (6)
Not stated	5 (2)
**Frequency of anal sex in the last year** *	
Several times a week	35 (14)
About once a week	89 (34)
About once a month	65 (25)
Less often than once a month	58 (22)
Not stated	13 (5)
**Frequency of vaginal sex in the last year** **	
Several times a week	0.8 (2)
About once a week	0.4 (1)
About once a month	0.8 (2)
Less often than once a month	5.8 (15)
None of the time	92.3 (240)
**Frequency of condom use in the last month**	
All the time	124 (48)
Most of the time	44 (17)
Some of the time	28 (11)
Rarely	7 (3)
None of the time	17 (7)
Not stated	40 (15)
**Transactional sex at present**	
Yes	31 (12)
No	229 (88)
**Injecting drug use risk factors – n (%)**	
Injecting drugs at present	
Yes	2 (1)
No	258 (99)

* Anal sex was insertive and/or receptive.

** Vaginal sex reported was bisexual.

Percentages may not total 100 because of rounding.

### Future Acceptability and Potential use of PrEP

As reported in Table 2, participants’ willingness to use PrEP (39.2% “yes, definitely” and 49.2% “yes, probably”) and to adopt it early (22.2% “yes, definitely” and 47.4% “yes, probably”) was high overall. Willingness to use PrEP remained high even after learning of potential mild side effects (24.6% “yes, definitely” and 56.5% “yes, probably”), having to pay 150 Baht/month for it (58.8% “yes, definitely” and 35.8% “yes, probably”), using a condom in combination with PrEP (52.7% “yes, definitely” and 36.9% “yes, probably”), or being regularly tested for HIV (43.6% “yes, definitely” and 44.8% “yes, probably”).

**Table 2:** Likelihood of PrEP use.

	Yes, definitely(%)	Yes, probably(%)	No, probablynot (%)	No, definitelynot (%)	Not stated^1^/Donot mind^2^ (%)
If PrEP became available, do you thinkyou would use it?	39.2	49.2	7.3	4.2	
Would you take PrEP as soon as itbecomes available?	19.6	41.9	25.8	1.2	11.5^1^
Would you take PrEP if it caused mildtemporary side effects?	24.6	56.5	13.8	5	
Would you take PrEP if you had to pay500 Baht a month for it?*	58.8	35	4.2	1.9	
Would you take PrEP even if you haveto use condoms?	52.7	36.9	8.5	1.9	
Do you think you would use PrEP if neededto be tested regularly for HIV/AIDS?	43.6	44.8	8.1	3.5	
Would you want your partner(s)to know that you are taking PrEP?	45.4	24.2	12.7	11.9	5.8^2^
Would you share PrEP if you only hadenough to protect yourself and was givenfor free?	26.5	43.8	12.7	16.9	
Would you sell PrEP to other peoplewho need it more than you?	6.9	28.8	20.4	43.5	
	**Very** **embarrassing**	**Fairly** **embarrassing**	**Not very** **embarrassing**	**Not at all** **embarrassing**	
How embarrassing would you find it totake PrEP?	2.7	5.8	15	76.5	
	**Very anxious**	**Fairly anxious**	**Not very anxious**	**Not at all anxious**	
How anxious does the thought of takingPrEP make you feel?	2.7	35.4	31.9	30	
	**A lot of hope**	**Some hope**	**Not much hope**	**No hope at all**	
How much hope does PrEP give you?	38.5	55.8	5	0.8	

* 150 Baht is equal to the cost of two packs of headache medicine in Thailand.

Percentages may not total 100 because of rounding.

We also found that a minority of participants would feel embarrassed about taking PrEP (2.7% “very embarrassing” and 5.8% “fairly embarrassing”) and most would want their partner or partners to know they were taking it (45.4% “yes, definitely” and 240.2% “yes, probably”). However, a considerable minority would feel anxious about taking PrEP (2.7% “very anxious” and 35.4% “fairly anxious”). Participants also reported intentions to share it (26.5% “yes, definitely” and 43.8% “yes, probably”) and sell it to those who needed it more (6.9% “yes, definitely” and 28.8% “yes, probably”). Yet, most participants felt that PrEP would give them hope for new possibilities in their lives (38.5% “a lot of hope” and 55.8% “some hope”).

### Participants’ Characteristics and Likelihood of PrEP Use

As reported in Table 3 and 4, willingness to take PrEP was positively associated with previous experience taking regular medication (U = 693, p<0.05, r = 0.16), fear of contracting HIV (rho = 0.15<0.05), having a greater number of sexual partners in the last month (rho = 0.13, p<0.05), and more frequent anal sex (rho = 0.13, p<0.05). Whilst willingness to take PrEP as soon as it becomes available was negatively associated with frequency of vaginal sex (rho = **-**0.16<0.05) and positively associated with frequency of anal sex (rho = 0.17<0.05). Willingness to take PrEP despite side effects (rho = 0.16, p<0.05), having to pay (rho = 0.16, p<0.01) and to use condoms (rho = 0.14, p<0.05) were positively associated with fear of contracting HIV.

**Table 3:** Correlations between participants’ characteristics and likelihood of PrEP use.

	Age	Education	Fear ofcontractingHIV	Number ofpartnersin the lastmonth	Frequency ofcondom usein the lastmonth	Frequency ofvaginal sexin the lastyear	Frequency ofanal sexin the lastyear
If PrEP became available, do you thinkyou would use it?	0.00	−0.02	**0.15** *	**0.13** *	0.02	−0.07	**0.13** *
Would you take PrEP as soon asit becomes available?	0.08	−0.05	0.09	0.02	−0.09	−**0.16** *	**0.17** *
Would you take PrEP if it causedmild temporary side effects?	0.08	−0.05	**0.16** *	0.10	−0.02	−0.07	0.08
Would you take PrEP if you hadto pay 500 Baht a month for it?*	0.02	0.01	**0.16** **	**0.16** *	−0.08	−**0.19** **	**0.20** **
Would you take PrEP even if you haveto use condoms?	−0.09	0.04	**0.14** *	0.11	0.03	0.02	0.10
Do you think you would use PrEPif needed to be tested regularlyfor HIV/AIDS?	−0.03	0.05	0.10	−0.03	0.04	−0.03	0.08
Would you want your partner(s)to know that you are taking PrEP?	−0.06	0.08	0.09	0.04	−**0.15** *	−0.01	**0.14** *
Would you share PrEP if you onlyhad enough to protect yourselfand was given for free?	−**0.13** *	−0.05	0.02	−0.05	0.01	0.00	0.04
Would you sell PrEP to otherpeople who need it more than you?	−**0.15** *	−**0.17** **	0.00	0.10	−0.10	−0.09	0.04
How embarrassing would youfind it to take PrEP?	0.04	−0.02	0.07	−**0.14** *	0.11	−0.03	−0.06
How anxious does the thoughtof taking PrEP make you feel?	−**0.13** *	−0.08	**0.15** *	−0.10	0.01	−**0.15** *	−0.09
How much hope does PrEPgive you?	0.02	−0.08	**0.22** **	**0.13** *	−0.12	−0.07	0.04

** Correlation is significant at the 0.01 level (2-tailed).

* Correlation is significant at the 0.05 level (2-tailed).

**Table 4:** Differences between participants’ characteristics and likelihood of PrEP use.

	City of residence				Experience taking regular medication				HIV testing				Transactional sex at present			
	MD	MD			MD	MD			MD	MD			MD	MD		
	B	C	*U*	*r*	Yes	No	*U*	*r*	Yes	No	*U*	*R*	Yes	No	*U*	*r*
**Willingness to take PrEP**	3	3	7815	0.07	3	3	693	0.16*	3	3	7511	0.08	4	3	3103	0.08
**As soon as it becomes available**	3	3	5639	0.14*	3.5	3	463	0.09	3	3	6051	0.06	3	3	2482	0.09
**Despite side effects**	3	3	7879	0.07	4	3	1194	0.02	3	3	7643	0.06	3	3	3061	0.09
**Despite having to pay**	4	4	7382	0.13*	4	4	1114	0.04	4	4	8098	0.01	4	4	3091	0.08
**Despite having to use condoms**	4	4	8227	0.03	4	3.5	1042	0.06	4	4	8075	0.01	4	4	2926	0.11
**Despite being tested regularly for HIV**	3	3	8231	0.02	3.5	3	908	0.10	3	3	7605	0.07	4	3	2527	**0.18** **
**Would you share PrEP**	3	3	8376	0.01	3	3	1063	0.05	3	3	7979	0.02	3	3	3309	0.04
**Would you sell PrEP**	2	1	7149	0.14*	1	2	903	0.03	2	2	7411	0.09	3	2	2508	**0.18** **
**Would want partner(s) to know**	4	4	8019	0.05	4.5	4	1035	0.15*	4	4	7263	0.10	4	4	2917	0.11
**Would find taking PrEP embarrassing**	1	1	8385	0.01	1	1	1059	0.07	1	1	7751	0.06	1	1	3491	0.01
**Would you feel anxious about taking PrEP**	2	2	8417	0.00	2.5	2	1091	0.04	2	2	7421	0.08	2	2	3102	0.07
**Would taking PrEP give you hope**	3	3	8360	0.01	4	3	1017	0.07	3	3	8179	0.00	4	3	2986	0.10

** Difference is significant at the 0.01 level (2-tailed). *Correlation is significant at the 0.05 level (2-tailed).

MD: median. U: Mann Whitney’s *U*-statistic. *r*: effect size estimate (*r* = *Z*/√N; N = number of observations). B: Bangkok; C: Chiang Mai.

Feeling embarrassed about taking PrEP was negatively associated number of partners in the last month (rho = −0.14, p<0.05), whereas feeling anxious about taking PrEP was negatively associated with age (rho = −0.13, p<0.05) and frequency of vaginal sex (rho = −0.15, p<0.05), and positively associated with fear of contracting HIV (rho = 0.15, p<0.05). Those who felt that PrEP would give them hope were more likely to be afraid of contracting HIV (rho = 0.22, p<0.01) and to have a greater number of sexual partners in the last month (rho = 0.13, p<0.05).

Reported intention to share PrEP was negatively correlated with age (rho = −0.13<0.05) and Intention to sell PrEP was negatively correlated with age (rho = −0.15<0.05) and education (rho = −0.17<0.01), and positively correlated with transactional sex (U = 2508, p<0.01, r = 0.18).

Participants living in Bangkok were more likely to take PrEP as soon as it becomes available (U = 5639, p<0.05, r = 0.14) and to pay for PrEP (U = 7382, p<0.05, r = −20.03), and less likely to sell PrEP (U = 7382, p<0.05, r = 0.13) than those living in Chiang Mai.

We found no association between frequency of condom use and frequency of anal sex in the last year and number of partners in the last month.

### Relative Importance of PrEP Hypothetical Attributes

**Figure 1:**
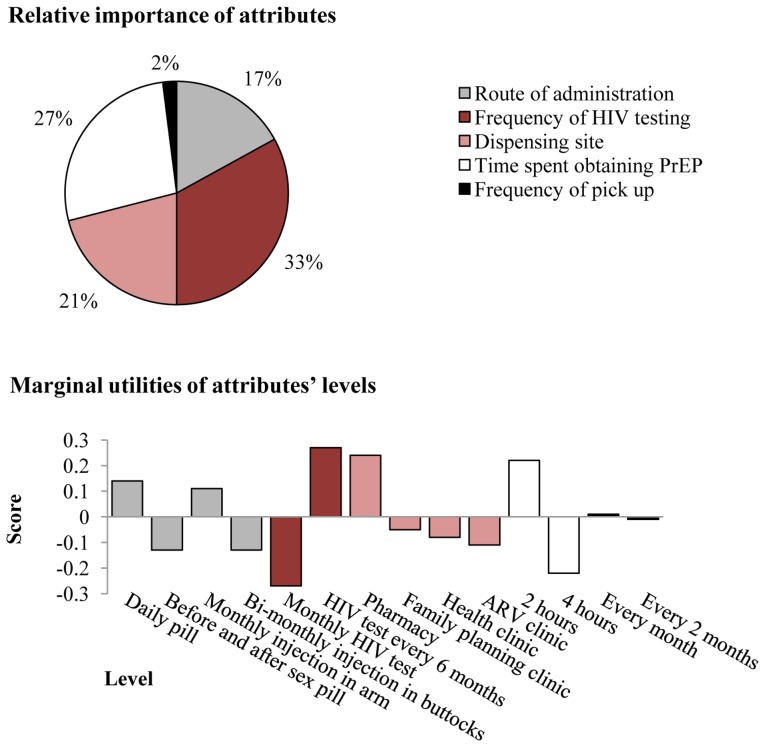
Relative importance of PrEP attributes and marginal utilities* of attributes’ levels. .3, −.3 is the Point Estimate of Alpha interval. *Conjoint analysis decomposes participants’ ranking of each scenario into the sum of contributions of the different PrEP attributes. Marginal utilities are the part-worth of a specific attribute in participants’ ranking of the scenarios. In other words, marginal utilities signify the extent to which a specific attribute contributes to the ranking of a scenario.

The frequency of HIV testing was the most important attribute of a PrEP program, followed by the time it would take to receive PrEP (Fig. 1). A daily pill was the preferred level of the route of administration, followed by a monthly injection in the arm. Yet only 4% of participants reported ever taking regular medication. The preferred frequency of testing was every six months. Pharmacies were the favored dispensing site. Frequency of pick up was not an influential determinant of PrEP use (Fig. 1).

## Discussion

Trial results have demonstrated that PrEP can be an effective prevention method against HIV. Yet PrEP acceptability and users’ ability to adhere to it are likely to have a significant impact on the success of this new initiative [38].

We examined Thai MSM’s attitudes and preferences towards PrEP to evaluate its future acceptability among this population. Our results show that most participants would consider taking PrEP despite its disadvantages. We found that participants living in Bangkok were more willing to take PrEP than those living in Chiang Mai, which may be explained by a more adverse epidemiological context. Interestingly, when participants were asked whether they would take PrEP even when having to pay an affordable amount, using condoms in combination with PrEP or being regularly tested for HIV, the majority reported they would. This indicates participants’ motivation to overcome barriers that can have a significant impact on PrEP uptake. It should not be inferred that willingness to take PrEP would increase in the presence of these particular barriers.

We found that participants who took regular medication in the past, were afraid of contracting HIV and reported having more partners and more frequent anal sex were also more willing to take PrEP. Our results also indicate that frequency of condom use was not associated with sexual risk behaviors. These findings suggest that those at risk of contracting HIV through sexual contact and importantly, perceive themselves to be at higher risk, were more motivated to enroll. Efforts should thus be focused on reaching these individuals and providing them with adequate information and access to available HIV prevention methods, including PrEP. Such efforts should include migrant populations who are currently not entitled to access public HIV prevention and treatment services [39].

Worryingly, younger and less educated participants – a proxy for socioeconomic status [35] –, and those engaging in transactional sex, were more likely to sell PrEP. Enhancing the quality of the communication between PrEP users and providers, stressing the risks of poor adherence and implementing relevant monitoring strategies will therefore be necessary to improve PrEP effectiveness in this demographic group.

Our results have implications for PrEP eventual demonstration projects in Thailand. Our data indicate that PrEP could be offered to MSM at higher risk in the first instance. PrEP should be affordable and implicit costs such as waiting times should be low. Delivering PrEP in a confidential and accessible healthcare setting would be desirable to reduce anxiety. A daily pill and a monthly injection in the arm would be acceptable routes of administration, which is encouraging from a policy perspective, as it would allow both a rapid implementation of existing oral PrEP and the eventual rollout of parenteral PrEP. The latter may reduce users' likelihood of sharing, selling or forgetting to take PrEP. In the meantime, monitoring measures and behavioral interventions would have to be put in place to ensure adequate levels of adherence to oral PrEP. Important lessons can be drawn from successful ART interventions [40], [41]. Further research to understand why the frequency of HIV testing was regarded as the most important PrEP attribute is needed to prevent it from becoming a barrier to implementation.

Reassuringly, the characteristics of our sample compare well to those of larger studies among MSM in Thailand [42], [43], making it more likely that the views of the participants of our study are representative of the assessed population. PrEP acceptability levels among Thai MSM resonate with those reported in previous research [20], [21], [23], yet drawing comparisons with these studies should be done with caution, as their methods differ greatly. Notably, Indian, South African and, to a lesser extent, Peruvian MSM reported willingness to use PrEP in an analogous study conducted by our team before the publication of the iPrEx results on the acceptability of mostly uncertain PrEP attributes [19]. This may indicate that the acceptability of a more realistic PrEP may be comparable to that of a largely hypothetical one. However, the same study shows that most MSM preferred parenteral PrEP, whereas Thai MSM preferred daily oral PrEP. This suggests that an existing but less convenient PrEP regimen may be preferred to a less demanding but hypothetical one. It may also indicate that Thai MSM may be overestimating their capacity to adhere to oral PrEP, as most participants reported not having experienced taking regular medication in the past.

There are some limitations to this study. Given the sensitive nature of the addressed questions, some participants may have felt at times inclined to provide what they felt was the “right” answer. Additionally, our data collection took place in urban areas, where HIV incidence is normally higher, thus current findings may not be generalizable to rural settings. Finally, examining acceptability among users enrolled in demonstration projects is much deserving, as actual acceptability may differ from potential willingness to take PrEP.

Our results offer valuable insights that can help to deliver PrEP more effectively, should Thailand decide to implement it, by focusing their efforts on the most critical aspects of its implementation. Importantly, offering combination prevention packages which encompass both behavioral and biomedical HIV prevention methods, depending on individual circumstances and needs, will be essential to control the spread of HIV [44]. Communicating PrEP benefits and disadvantages in an unbiased and concise manner and involving key community representatives in PrEP implementation process will help to reduce potential apprehensions among stakeholders, facilitate PrEP introduction and increase uptake [45]. Yet, tackling stigma and healthcare inequalities will be necessary for existing and new prevention methods to significantly decrease HIV incidence among high-risk populations.
